# Calcium-Triggered PuMYB44-PuERF118 Cascade Activates *PuFAD8* to Promote Aroma Biosynthesis in Pear Fruit

**DOI:** 10.3390/ijms27188070

**Published:** 2026-09-10

**Authors:** Xinyu Wang, Gaifang Yao, Min Zhang, Huihui Song, Zhen Cao, Kangdi Hu, Xinxin Gai, Hua Zhang, Gaihua Qin

**Affiliations:** 1 Key Laboratory of Germplasm Innovation and Utilization of Horticultural Crop (Co-Construction by Ministry and Province), Ministry of Agriculture and Rural Affairs, Institute of Horticultural Research, Anhui Academy of Agricultural Sciences, Hefei 230031, China; 2 School of Food and Biological Engineering, Hefei University of Technology, Hefei 230009, China; yaogaifang@hfut.edu.cn (G.Y.); kangdihu@hfut.edu.cn (K.H.);

**Keywords:** pear (*Pyrus ussuriensis*), aroma, PuMYB44-PuERF118, PuFAD8, transcriptional regulation

## Abstract

Fruit aroma is a key factor shaping the commercial value of pears. MYB and ERF transcription factors (TFs) are involved in regulating the biosynthesis of fruit aroma compounds, yet the mechanisms underlying their synergistic regulation of pear aroma formation remain elusive. Calcium signaling exerts an important role in fruit aroma biosynthesis; nevertheless, how calcium signaling mediates MYB-ERF transcription factors to participate in pear aroma biosynthesis remains unclear. Here, transcriptomic profiling of calcium-treated pear fruits screened two significantly upregulated TFs, PuMYB44 and PuERF118, as candidate genes. Phylogenetic analysis and conserved domain characterization further suggested that these two genes may be involved in regulating fatty acid biosynthesis. Transient overexpression experiments in pear fruits revealed that the co-expression of PuMYB44 and PuERF118 synergistically activated a set of fatty acid biosynthesis genes, including *PuFAD2*, *PuFAD3*, *PuFAD8*, *PuLOX5*, *PuLOX21*, and *PuSCD*. Meanwhile, the co-expression markedly increased the contents of key aroma volatiles, such as ethyl acetate, 1-hexanol, ethyl 2-methylbutyrate and hexadecane. Yeast two-hybrid and luciferase complementation assays confirmed an interaction between PuMYB44 and PuERF118. Analysis of the PuFAD8 promoter sequence revealed a conserved MYB-binding motif and an ERF-responsive cis-acting element. Dual-luciferase reporter assays further confirmed that both PuMYB44 and PuERF118 can independently activate *PuFAD8* transcription, and that the synergistic activation effect resulting from the co-expression of these two factors is significantly stronger than that observed when either factor is expressed alone. In summary, this study elucidated a calcium-responsive transcriptional module underlying the biosynthesis of pear aroma and provided potential molecular targets for improving the flavor quality of pear fruit.

## 1. Introduction

Pear (*Pyrus ussuriensis*) is one of the world’s most economically important fruit crops and is widely loved by consumers for its unique taste and aroma. Aroma directly determines the market value of pears and consumers’ purchasing preferences, and it also serves as an important attribute for evaluating fruit quality [1]. Fruit aroma is composed of specific volatile organic compounds (VOCs), which are primarily derived from fatty acid metabolism, terpenoid biosynthesis, and amino acid metabolic pathways [2,3]. Among these pathways, esters and aldehydes synthesized via the lipoxygenase (LOX) pathway are the primary contributors to the unique aroma in pear fruits [4]. Key enzymes in this metabolic pathway include fatty acid desaturases (FADs) and lipoxygenases (LOXs), which catalyze the conversion of linoleic acid and linolenic acid into volatile precursors and further convert these precursors into aroma-active esters, thereby forming the unique fruit aroma [5].

Fatty acid desaturases (FADs) modulate the substrate availability for unsaturated fatty acid synthesis by introducing carbon–carbon double bonds [6,7]. In recent years, accumulating studies have revealed a close correlation between the expression levels of FAD genes and fruit aroma quality. For instance, silencing the SlFAD7 gene in tomato significantly reduces the accumulation of C6 volatiles, confirming that mitochondria-localized FADs are essential for the biosynthesis of green-note aroma compounds [8]. In peach fruits, the differential expression of the PpFAD genes during ripening is associated with dynamic changes in fatty acid composition and the accumulation of characteristic lactones and esters [9]. Several PuFAD genes have been identified in pears, and studies have shown that the transcriptional levels of FAD genes are positively correlated with the release of key aroma compounds [10,11]. These findings suggest that FADs are key regulators of fruit aroma biosynthesis; however, research on the upstream transcription factors that regulate FAD expression and coordinate their interaction with other metabolic pathways remains limited.

Fruit aroma biosynthesis is governed by a complex regulatory network involving multiple transcription factors. Accumulating evidence has demonstrated that MYB, ERF, and WRKY families play key roles in regulating aroma formation [12]. These transcription factors often regulate aroma biosynthesis by synergistically controlling the transcription of downstream structural genes. Among them, MYBs are widely reported to participate in multiple biological processes, including secondary metabolism, organ morphogenesis, and circadian rhythms [13,14]. In particular, R2R3-MYB transcription factors play a crucial role in fruit aroma biosynthesis. In apples, *MdMYB31* promotes the accumulation of volatile esters by directly binding to and activating the promoters of the key ester synthase genes *MdAAT1* and *MdAAT2* [15]. MYB transcription factors can also regulate aroma compounds by forming complexes; for example, *LiMYB1* in lilies interacts with *LiMYB308* and LiMYB330 to synergistically regulate the biosynthesis of lily terpenoids [16].

In addition, fruit traits often change synchronously during development, particularly color and aroma, suggesting that there may be interactions or transcriptional regulatory mechanisms among different regulatory pathways [17]. MYB transcription factors play important regulatory roles in both the anthocyanin biosynthesis pathway and the biosynthesis pathway of aromatic compounds [18]. For example, *FhMYB4* in the tigerlily (*Lilium tigrinum*) binds to MYB cis-acting elements in the *FhDFR3* and *FhTPS1* promoters, thereby inhibiting the synthesis of linalool; simultaneously, it interacts with FhTT8L to disrupt the MYB-bHLH-WD40 (MBW) activation complex associated with anthocyanins, thereby inhibiting anthocyanin biosynthesis [18]. In roses, the anthocyanin-related *RcMYB1* gene is also involved in regulating the synthesis of carotenoids and volatile aromatic metabolites [19]. Overexpression of *Arabidopsis AtPAP1* in hybrid roses simultaneously enhances the production of phenylpropanoid pigments and terpenoid aromatic compounds [20].

Ethylene response factors (ERFs) are involved in ethylene signal transduction and play a crucial role in regulating plant growth and helping plants withstand stress [21]. Recent studies have shown that ERFs are associated with flavor changes during fruit ripening [22]. In apples, *MdERF74* and *MdERF75* regulate the synthesis of aroma compounds through fatty acid metabolic pathways [23]. *PpERF5* and *PpERF7* can interact to form a complex that upregulates the expression of *PpLOX4*, thereby enhancing peach aroma [24]. *PpERF61* regulates the linalool biosynthesis genes *PpTPS1* and *PpTPS3* during peach fruit ripening, thereby promoting peach aroma biosynthesis [25]. Previous studies have reported that MYB and ERF proteins regulate the biosynthesis of fruit aroma compounds by forming MYB-ERF complexes. For example, in strawberries, FaERF9 forms a complex with FaMYB98, thereby activating the FaQR promoter and promoting the biosynthesis of HDMF in strawberries [26].

Calcium (Ca^2+^) serves as a ubiquitous second messenger in plant cells, mediating a wide range of physiological processes, including fruit ripening and stress signal transduction [27]. Recent studies have shown that calcium supplementation can effectively alter aroma characteristics of fleshy fruits, possibly by regulating the expression of specific regulatory transcription factors [28]. However, the precise molecular mechanisms by which calcium-responsive transcription factors regulate fatty acid metabolic pathways remain unclear.

In this study, heatmap and bioinformatics analyses of transcriptomic data from calcium-treated pear fruits were performed to screen and identify two candidate transcription factors, *PuMYB44* (Pbr025199.1) and *PuERF118* (Pbr028836.1). The expression patterns of these two genes were further validated in ‘Daxiangshui’ and ‘Hwangkeum’ pear fruits by qRT-PCR. Transient overexpression assays further demonstrated that co-transformation of *PuMYB44* and *PuERF118* significantly induces the biosynthesis of aroma compounds and markedly upregulates the expression of fatty acid desaturase (*FAD*) family genes in pear fruit. In vitro protein interaction assays further verified the physical interaction between PuMYB44 and PuERF118. Heatmap and correlation analyses indicated that *PuFAD8* (Pbr005591.1) exhibits a stronger correlation with *PuMYB44* and *PuERF118* than other homologous genes. Further promoter sequence analysis and dual-luciferase reporter assays confirmed that PuMYB44 and PuERF118 can activate the *PuFAD8* promoter by binding to MYB and ERF cis-acting elements, respectively. Moreover, their co-expression exerts a significantly stronger transcriptional activation effect on *PuFAD8* than the individual expression of either gene. This study provides novel molecular targets for the flavor quality improvement in pear fruit.

## 2. Results

### 2.1. PuMYB44 and PuERF118 Were Identified as Transcriptional Regulators of Aroma Compound Biosynthesis

**Figure 1:**
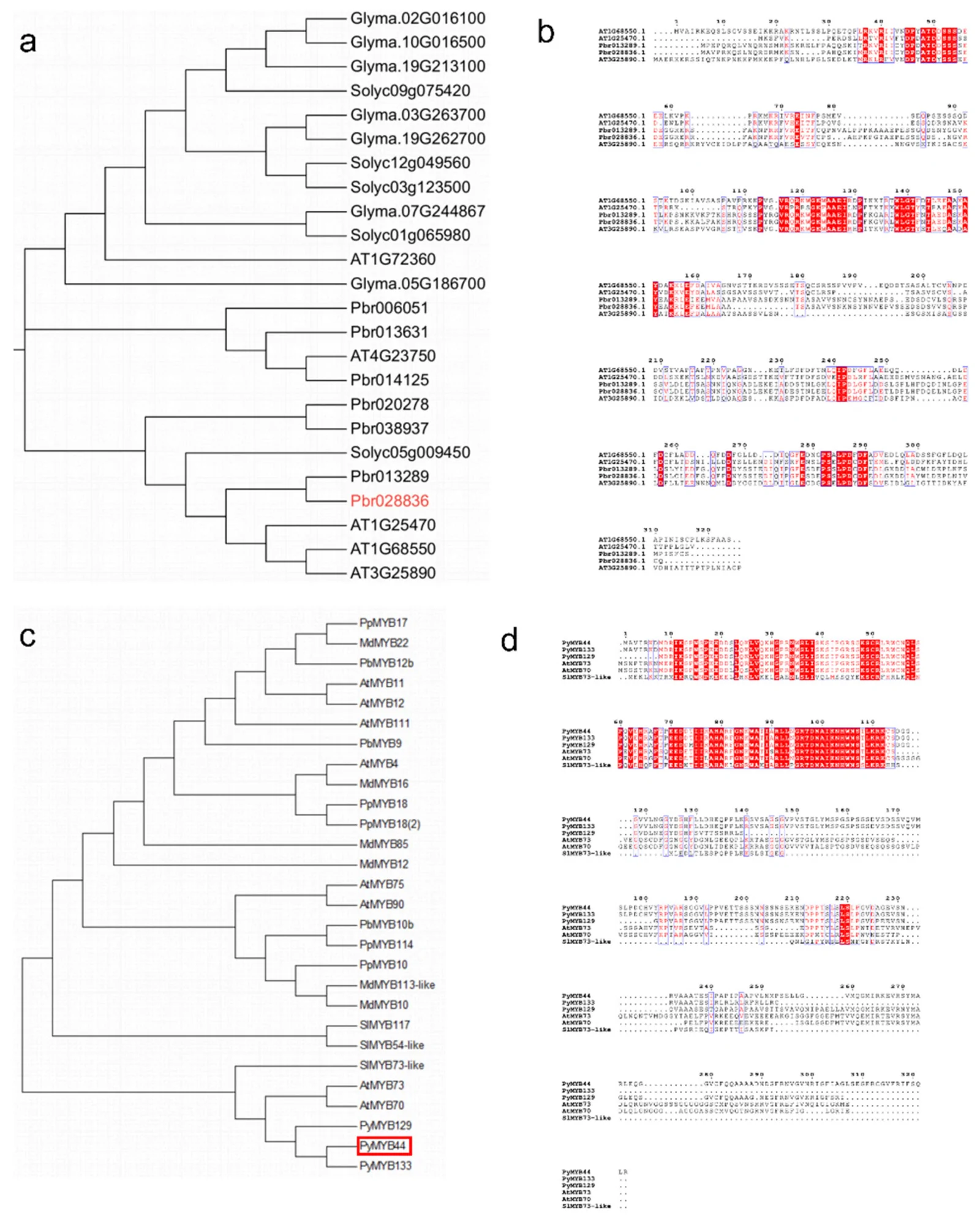
Phylogenetic analysis, sequence alignment of *PuMYB44* and *PuERF118* and homologues in different plant species. (**a**) Phylogenetic tree analysis of *PuMYB44*. The full-length amino acid sequences were aligned using ClustalW. The phylogenetic tree was generated using MEGA7 software. (**b**) Multiple sequence alignment of *PuMYB44* proteins across plant species. The consensus subdomains in the *PuMYB44* binding domain are shown by blue lines. (**c**) Phylogenetic tree analysis of *PuERF118*. The full-length amino acid sequences were aligned using ClustalW. The phylogenetic tree was generated using MEGA7 software. (**d**) Multiple sequence alignment of *PuERF118* proteins across plant species. The consensus subdomains in the *PuERF118* binding domain are shown by blue lines.

From transcriptomic datasets generated from calcium-treated pear fruits at sequential developmental stages [29], we identified two transcription factors, PuMYB44 (Pbr025199.1) and PuERF118 (Pbr028836.1), whose expression was consistently induced by CaCl_2_ treatment. Z-score-normalized heatmap analysis placed these two genes within a cluster of strongly upregulated transcripts, pointing to their potential involvement in calcium-mediated regulatory networks (Figure S1). To gain insights into the evolutionary relationships and structural features of PuMYB44 and PuERF118, we conducted phylogenetic reconstruction and multiple sequence alignment with homologous proteins from pear, apple, peach, *Arabidopsis*, and tomato. For PuMYB44, the neighbor-joining (NJ) tree placed it closest to pear PuMYB113, with which it shares a highly conserved N-terminal R2R3 DNA-binding domain. In contrast, the C-terminal regions exhibit considerable divergence, suggesting functional specialization across species (Figure 1a,b). Similarly, phylogenetic analysis of PuERF118 assigned it to a clade containing Arabidopsis *AT1G25470* and *AT1G68550*, and multiple sequence alignment revealed a strictly conserved core AP2/ERF domain across all orthologs, with key residues and structural motifs marked in red and blue, respectively (Figure 1c,d). Collectively, the phylogenetic proximity of PuMYB44 and PuERF118 to functionally characterized regulators of aroma metabolism, together with the retention of their canonical DNA-binding domains, points to a potential role for these two transcription factors in modulating volatile compound biosynthesis in pear.

### 2.2. Expression Profiles and Correlation Analysis of Aroma-Related Transcription Factors in ‘Hwangkeum’ and ‘Daxiangshui’ Pear Varieties

**Figure 2:**
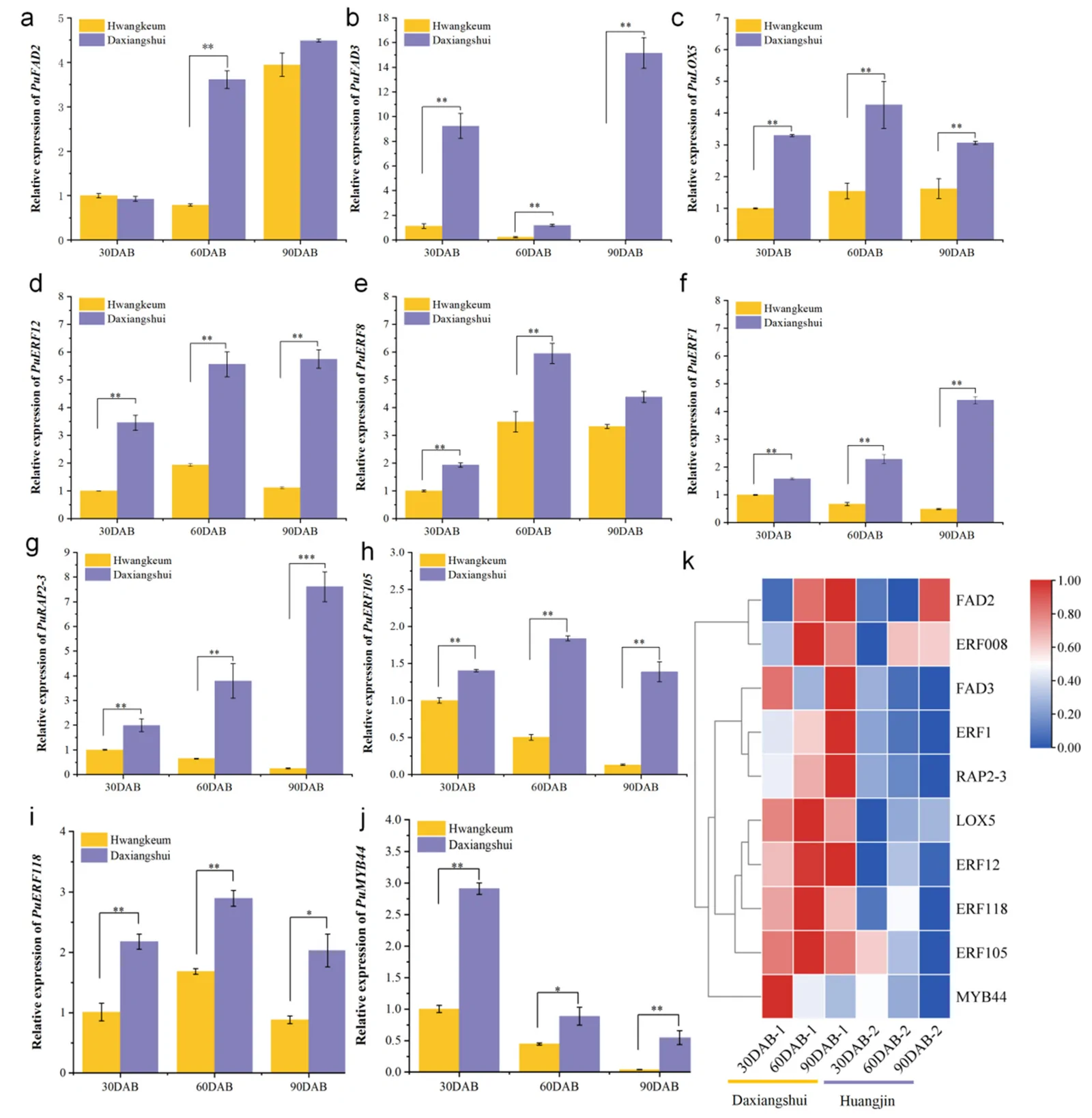
Analysis of the Expression Patterns and Correlations of Aroma-Synthesizing Genes in ‘Hwangkeum’ and ‘Daxiangshui’ Pears at Different Stages of Fruit Development. (**a**–**j**) Real-time quantitative fluorescent PCR (qRT-PCR) was used to detect the relative expression levels of *PuFAD2*, *PuFAD3*, *PuLOX5*, *PuAP2-3*, *PuERF105*, *PuERF12*, *PuERF8*, *PuERF1*, *PuERF118*, and *PuMYB44* in the fruits of the two pear varieties at 30, 60, and 90 DAB after flowering. Yellow and purple bar charts represent the ‘Hwangkeum’ and ‘Daxiangshui’ varieties, respectively. Data are presented as the mean ± standard deviation (SD) of three biological replicates. Asterisks indicate significant differences determined by Student’s *t*-test (* *p* < 0.05, ** *p* < 0.01, *** *p* < 0.001). (**k**) Hierarchical clustering heatmap of relative gene expression levels between ‘Hwangkeum’ and ‘Daxiangshui’ at 30, 60, and 90 DAB. The color scale (0 to 1.00) represents relative expression abundance, with red to blue indicating expression levels from high to low. Gene expression values have been Z-score normalized by row.

‘Daxiangshui’ is known for its rich aroma, while ‘Hwangkeum’ has a mild aroma; this is due to differences in the expression of aroma-related genes between the two varieties [30,31]. To profile the expression dynamics of aroma-associated genes during fruit maturation, we examined the transcript levels of PuMYB44, PuERF118, and additional candidate genes in ‘Hwangkeum’ and ‘Daxiangshui’ pear fruits at 30, 60, and 90 days after blooming (DAB) via qRT-PCR (Figure 2a–j). Notably, at all developmental stages studied, the expression levels of *PuMYB44* and *PuERF118* in ‘Daxiangshui’ were significantly higher than those in ‘Hwangkeum’ pear (Figure 2i,j). In particular, *PuERF118* showed a particularly significant upregulation at 30 days after blooming (*p* < 0.01), suggesting that these genes may be involved in enhancing the aromatic characteristics of the ‘Daxiangshui’ variety. To further investigate the relationship between the two, hierarchical cluster analysis revealed that *PuMYB44* and *PuERF118* were clustered within the ‘Daxiangshui’ high-expression gene set (Figure 2k). Furthermore, Pearson correlation analysis indicated a significant positive correlation between *PuMYB44* and *PuERF118* (*r* = 0.60), as well as significant associations with other aroma biosynthesis-related genes such as *PuLOX5* and *PuERF1* (Figure S2). Together, these results suggest that *PuMYB44* and *PuERF118* contribute to the biosynthesis of aromatic volatiles in pear fruit.

### 2.3. Transient Overexpression of PuMYB44 and PuERF118 Activates Aroma Biosynthesis-Related Genes

**Figure 3:**
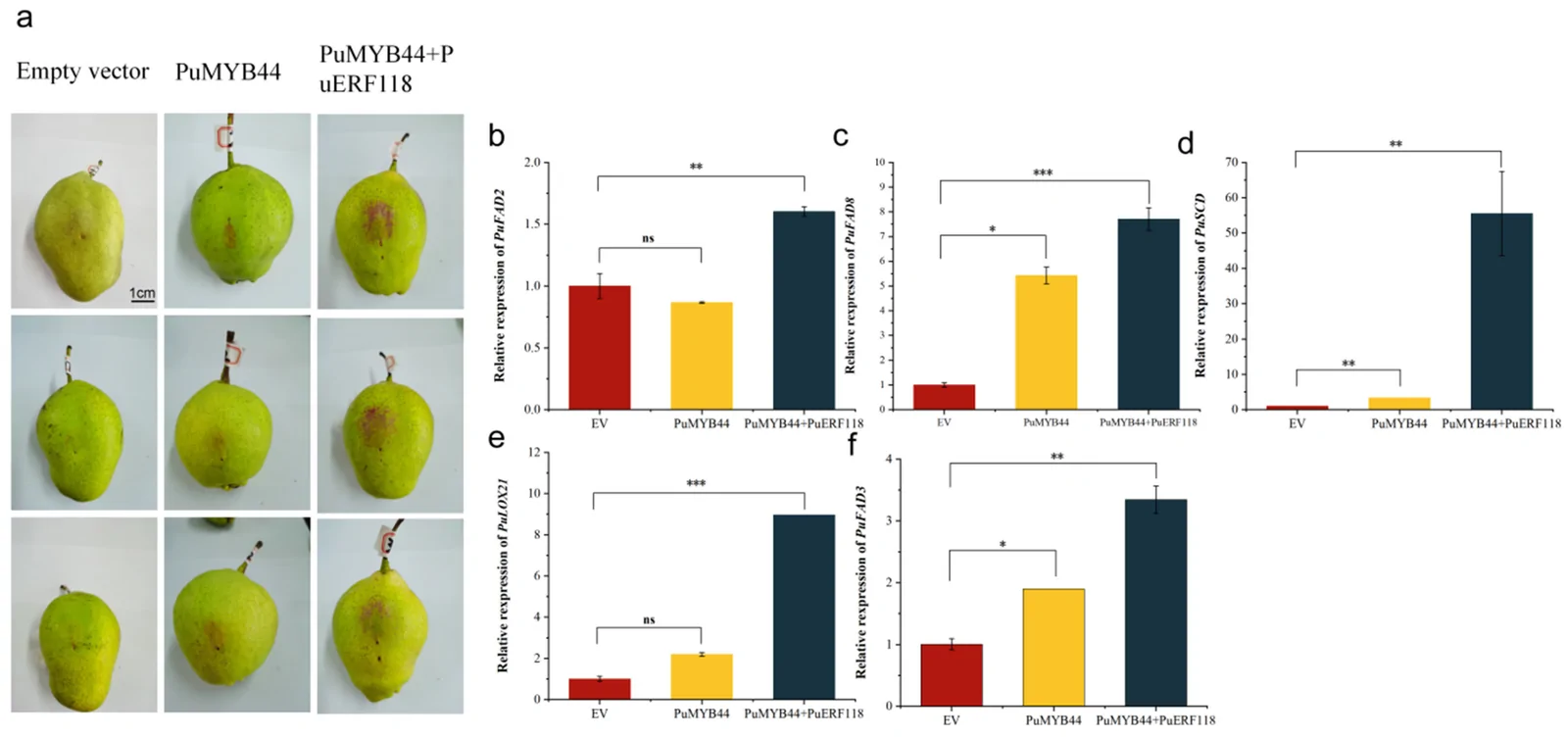
Effects of transient overexpression of PuMYB44 and PuERF118 on anthocyanin accumulation and the expression of related genes in pear fruits. (**a**) Phenotypic observations of pear fruits following injection with an empty vector, PuMYB44, or PuMYB44 co-expressed with PuERF118. After injection, varying degrees of color changes appeared on the fruit epidermis. (**b**–**f**) Relative expression levels of key structural genes downstream of the fatty acid synthesis pathway (*PuFAD2*, *PuFAD3*, *PuFAD8*, *PuSCD*, *PuLOX21*). Data are presented as mean ± standard deviation. “ns” indicates no significant difference, * *p* < 0.05, ** *p* < 0.01, and *** *p* < 0.001 levels, respectively (ANOVA combined with Duncan’s multiple range test).

Transient overexpression of PuMYB44 alone induced conspicuous red pigmentation on the fruit epidermis, and co-expression with PuERF118 further enhanced this coloration substantially, suggesting that *PuMYB44* and *PuERF118* also play important roles in the anthocyanin biosynthesis pathway (Figure 3a). Upon conducting a quantitative analysis of gene expression levels involved in the biosynthesis of aromatic compounds, we found that although the overexpression of PuMYB44 alone significantly upregulated the expression of PuFAD8, PuSCD, and PuFAD3, it had no significant effect on the transcriptional levels of PuFAD2 and PuLOX21. In stark contrast, the co-expression of PuERF118 and PuMYB44 produced a synergistic activation effect, inducing the expression of all structural genes, including PuFAD2 and PuLOX21 (Figure 3b–f). Collectively, these results demonstrate that PuMYB44 and PuERF118 act synergistically to activate downstream fatty acid pathway genes, thereby promoting the biosynthesis of volatile aroma compounds in pear fruit.

### 2.4. Volatile Profiling and Expression Correlation Analysis Identify PuFAD8 as a Key Candidate Gene for Pear Aroma Biosynthesis

**Figure 4:**
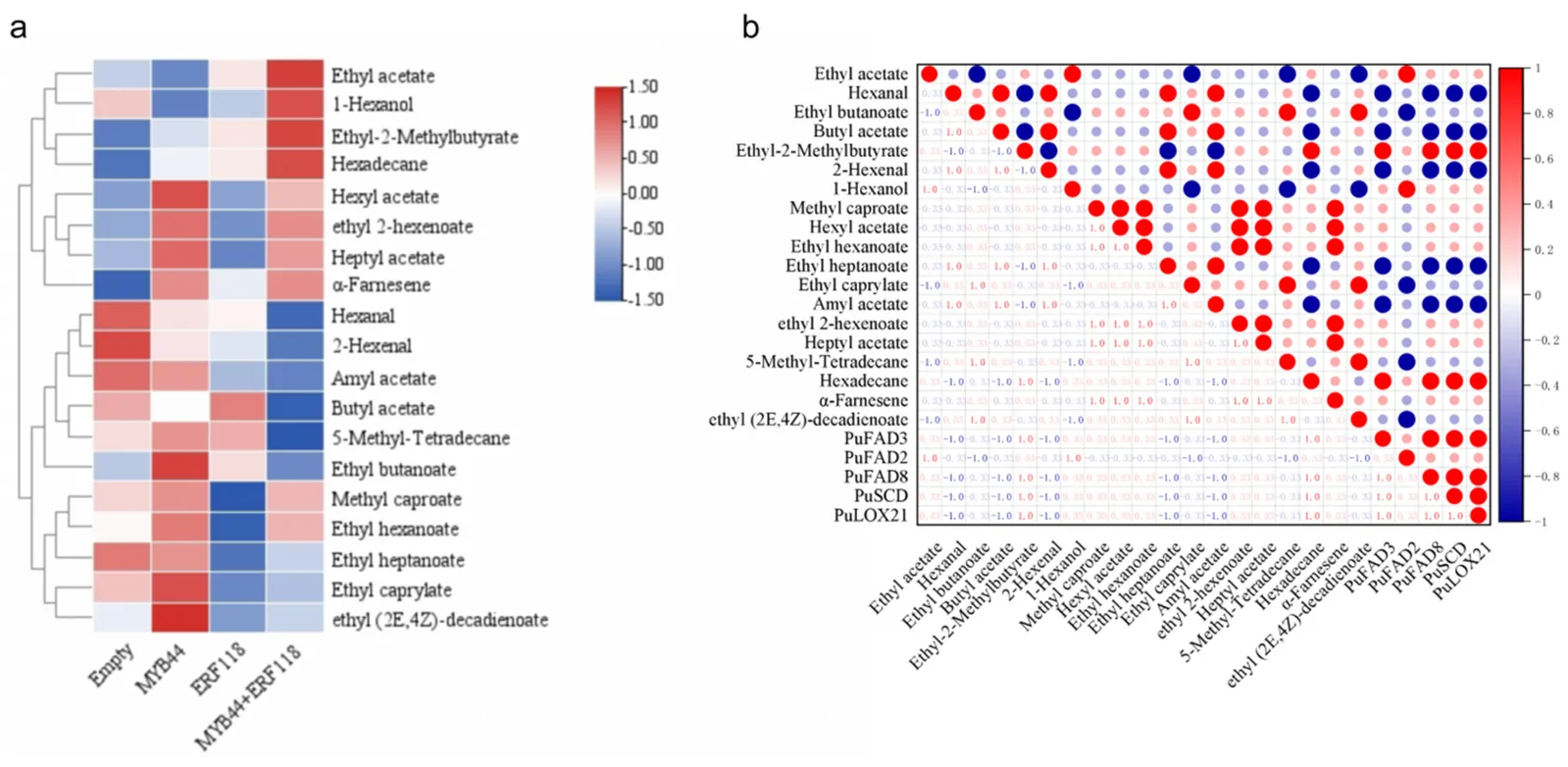
Correlation between volatile aroma compounds and structural genes in pear fruits, and metabolite accumulation patterns under different treatments. (**a**) Clustering heatmap of the relative contents of various volatile flavor compounds under different transient expression treatments (Empty, PuMYB44, PuERF118, PuMYB44 + PuERF118). The color scale (–1.50 to 1.50) indicates the relative abundance of compounds normalized by row; red indicates high accumulation, and blue indicates low accumulation. (**b**) Heatmap showing the correlation between volatile aroma compounds and the expression of genes related to lipoxygenase synthesis. The numbers and color intensity in the figure represent the Pearson correlation coefficient (r value); the red-to-blue gradient indicates a transition from positive to negative correlation, and significant correlations are indicated by the size of the circles.

We analyzed the volatile compound profiles in pear fruits treated with an empty vector, PuMYB44 alone, or a combination of PuMYB44 and PuERF118 to evaluate the metabolic outcomes of co-expression of these transcription factors. In the co-expression group, the accumulation levels of C6 aldehydes ethyl acetate, 1-hexanol, ethyl 2-methylbutyrate and hexadecane were consistently the highest, whereas the levels in the empty vector control group were the lowest (Figure 4a). Pearson correlation analysis indicated that *PuFAD8* transcript abundance showed the most significant correlation with these upregulated volatiles, with correlation coefficients exceeding 0.85 in most cases; this pattern was not observed in the other fatty acid genes tested (Figure 4b). This unique correlation pattern led us to hypothesize that *PuFAD8* may function as a key gene regulating the synthesis of aromatic compounds under the influence of PuMYB44 and PuERF118, prompting us to further investigate whether these two transcription factors directly act on the *PuFAD8* promoter.

### 2.5. PuMYB44 and PuERF118 Interact to Jointly Activate the Transcription of PuFAD8

**Figure 5:**
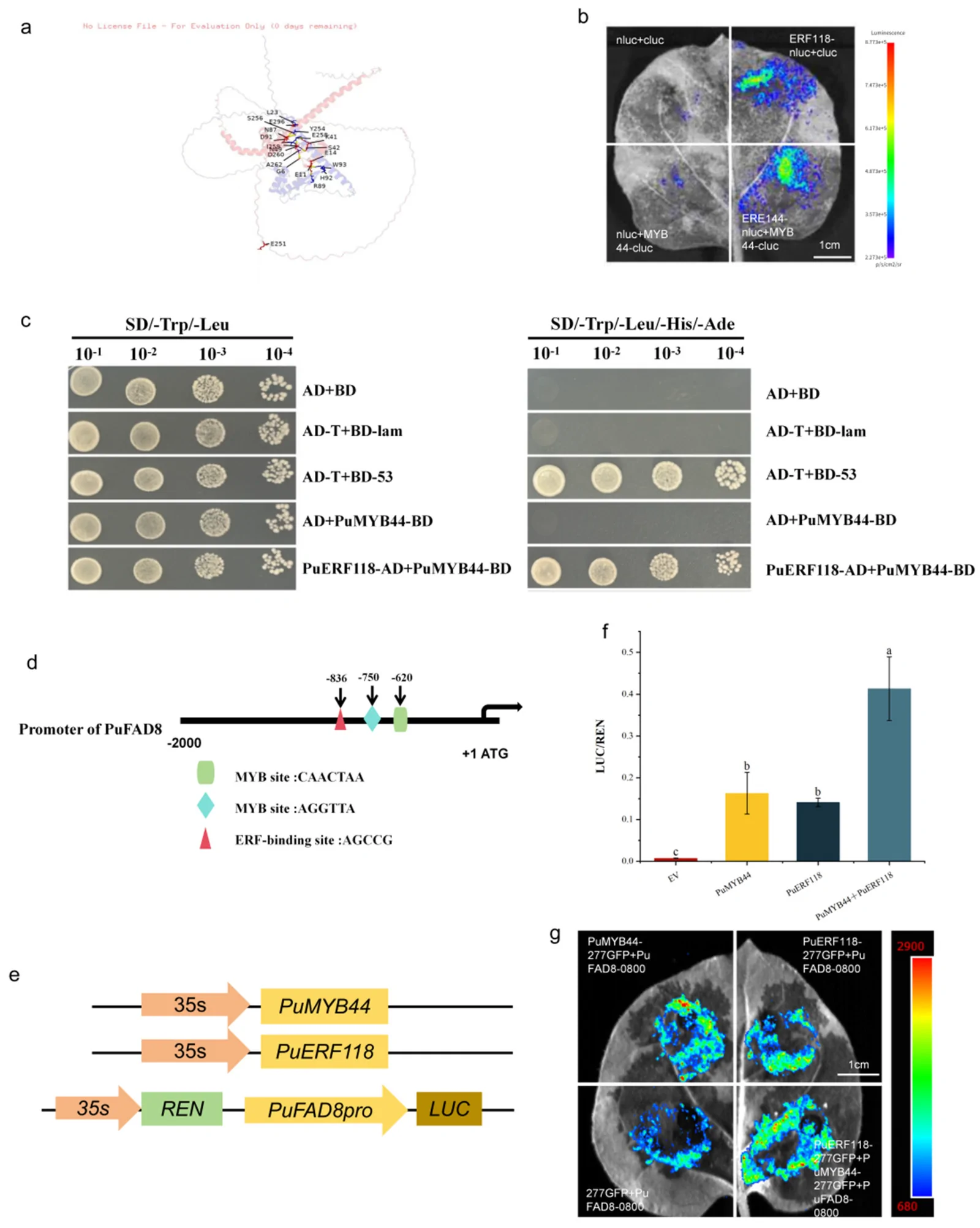
PuMYB44 and PuERF118 synergistically activate the PuFAD8 promoter. (**a**) Predicted molecular docking models of PuERF118 (red) and PuMYB44 (blue). The side chains of key residues are represented as stick models. The core amino acid residues involved in this interaction are clearly labeled, highlighting the key molecular recognition interface between PuMYB44 and PuERF118. (**b**) A firefly luciferase complementation imaging assay showing the interaction of PuMYB44 with PuERF118. Representative images from three independent biological replicates are shown. The color scale bar indicates the range of the luminescence intensity. (**c**) Yeast two-hybrid (Y2H) assay confirming the interaction between PuMYB44 and PuERF118. Yeast cells co-transformed with the indicated plasmid combinations were grown on SD/-Leu/-Trp (growth control) and SD/-Leu/-Trp/-His/-Ade (interaction selection) media. Cultures were spotted as four serial 10-fold dilutions (10^−1^ to 10^−4^). pGADT7-T/pGBKT7-53 and pGADT7-T/pGBKT7-Lam served as positive and negative controls, respectively; AD + PuMYB44-BD was included as a self-activation control. (**d**) Schematic diagram of the PuFAD8 promoter region. It includes the ERF binding site and two MYB binding sites, relative to the translation start codon (+1 ATG). (**e**) Assay using a dual firefly luciferase (DLR) reporter gene for transcription factor activation: Schematic diagram of the effector construct (expressing PuMYB44 and PuERF118) and the reporter construct containing the PuFAD8 promoter, which drives firefly luciferase (LUC) expression, with sea kidney luciferase (REN) serving as an internal control. Validate the transcriptional activation of PuFAD8 by candidate transcription factors using DLR assays. (**f**) A dual-luciferase reporter assay demonstrates the co-activator activity of PuMYB44 and PuERF118 on the PuFAD8 promoter. Data are presented as the mean ± standard deviation (or standard error) from three independent biological replicates. Different lowercase letters above the bar graphs indicate significant differences between treatment groups. (**g**) In vivo LUC luminescence imaging of the dual-luciferase reporter assay in *Nicotiana benthamiana* leaves. Agrobacterium suspensions carrying the PuFAD8 promoter-LUC reporter (PuFAD8-0800) were co-injected with the empty vector control (277GFP, EV), PuMYB44-277GFP alone, PuERF118-277GFP alone, or PuMYB44-277GFP together with PuERF118-277GFP into young tobacco leaves. LUC luminescence was imaged 48 h after injection. The false-color scale represents luminescence intensity (range, 680–2900). Scale bar = 1 cm. Co-injection of PuMYB44 and PuERF118 produced the strongest LUC signal, consistent with the quantitative LUC/REN results shown in (**f**).

To verify whether there is an interaction between PuMYB44 and PuERF118, AlphaFold3 was used to predict the likelihood of an interaction between the two, and PyMOL (3.1.6.1) software was used for visualization and analysis, revealing that the two proteins may form a complex through hydrogen bonds between multiple amino acids (Figure 5a). In the firefly luciferase complementation assay, although PuERF118-nluc exhibited some self-activation, a stronger fluorescent signal was still observed when PuERF118-nluc was co-expressed with PuMYB44-cluc (Figure 5b). We further validated the protein-level interaction between PuMYB44 and PuERF118 via yeast two-hybrid assays (Figure 5c). Therefore, we conclude that PuMYB44 and PuERF118 may participate in the regulatory network governing the synthesis of aromatic compounds as a complex. To investigate how the PuMYB44-PuERF118 protein complex participates in the regulation of aroma compound biosynthesis, we analyzed the promoter sequences of each of the aforementioned structural genes. We found that PuFAD8 promoter contains multiple transcription factor binding sites, including one ERF binding site (AGCCG) located at –836 bp and MYB binding motifs (AGGTTA and CAACTAA) located at –750 bp and –620 bp, respectively. We hypothesize that MYB and ERF may jointly participate in the regulation of *PuFAD8* transcription (Figure 5d). Dual-luciferase assays showed that, compared with the control group, the transcriptional activity of *PuFAD8* was significantly increased when PuMYB44 or PuERF118 was added alone, indicating that both factors can independently target and activate the *PuFAD8* promoter. Furthermore, the *PuFAD8* transcriptional activity resulting from the co-expression of PuMYB44 and PuERF118 was significantly higher than that observed when PuMYB44 or PuERF118 was injected alone (Figure 5e,f). In vivo LUC imaging of the injected tobacco leaves further corroborated these quantitative results: leaves co-injected with PuMYB44 and PuERF118 exhibited the strongest luminescence signal, whereas either factor alone produced a moderate signal and the empty-vector control showed only basal-level luminescence (Figure 5g). Overall, we have identified a molecular regulatory network in which PuMYB44 interacts with PuERF118 to synergistically activate the transcription of *PuFAD8*, thereby promoting the synthesis of aroma compounds in pears.

## 3. Discussion

Fruit aroma is a key quality indicator for pears, directly influencing consumer preferences and the commercial value of pears [3]. Therefore, studying the molecular mechanisms that regulate pear fruit aroma is of great significance. Although previous studies have reported that fatty acid metabolic pathways are the primary source of ester-type volatiles in pears [23,30], the transcriptional regulatory networks governing these pathways remain unclear.

As a second messenger widely present in plants, calcium plays a significant role in various physiological processes within the plant [31]. However, the relationship between calcium-signal-mediated biosynthesis of volatile compounds in fruit and the transcriptional regulatory networks remains unclear. In this study, analysis of transcriptomic data from pear fruits treated with 4% (*w*/*v*) calcium chloride revealed that calcium treatment induced differential expression of a series of genes, among which the transcription factors PuMYB44 and PuERF118 exhibited the most prominent changes in expression levels (Figure S1). Further analysis of the expression patterns of the aforementioned genes in the ‘Hwangkeum’ and ‘Daxiangshui’ pear varieties revealed that the expression levels of PuMYB44 and PuERF118 were significantly higher in ‘Daxiangshui’ pears at various developmental stages than in ‘Hwangkeum’ pears (Figure 2i,j). Results from transient co-transfection in pear cells demonstrated that the synergistic action of these two genes significantly promotes the expression of downstream structural genes related to aroma compound synthesis; yeast two-hybrid and luciferase complementation assays confirmed that PuMYB44 and PuERF118 interact at the protein level (Figure 5b,c). Previous studies have shown that MYB and ERF transcription factors in plants often function synergistically as complexes to regulate biological processes. For example, FaMYB98 and FaERF9 in strawberries form a protein complex that regulates aroma metabolism [26], while PpERF5 and PpERF7 in peaches participate in the regulation of volatile compound synthesis pathways by forming a heterodimer [24]. In Freesia hybrida, the AP2/ERF transcription factor FhAP2 physically interacts with the MYB activators FhPAP1 and FhMYB21L2, thereby disrupting the binding of these MYB regulators to their target gene promoters in a dose-dependent manner and coordinately repressing both anthocyanin and linalool biosynthesis, thus achieving coupled suppression of floral color and scent [32]. However, current research on MYB-ERF complexes regulating traits in pears remains primarily focused on the anthocyanin biosynthesis pathway, and reports on their functions in the biosynthesis of aroma compounds are still limited [33,34]. This study, through transient transformation experiments combined with gas chromatography-mass spectrometry (GC-MS) metabolite analysis, confirmed that the synergistic interaction between PuMYB44 and PuERF118 significantly promotes the accumulation of various aroma compounds, particularly ethyl acetate, 1-hexanol, ethyl 2-methylbutyrate, and hexadecane (Figure 4a). It is worth noting that aroma biosynthesis mechanisms may differ across Pyrus species. In ‘Nanguo’ pear, ester-type volatiles derived from the fatty acid pathway predominate, as well as, as shown in the present study, the PuMYB44-PuERF118-PuFAD8 module. By contrast, in Pyrus pyrifolia cultivars such as ‘Hwangkeum’ and ‘Daxiangshui’, the aroma profiles differ quantitatively, with ‘Daxiangshui’ exhibiting richer volatile emission that correlates with higher expression of PuMYB44 and PuERF118. These cross-species comparisons suggest that MYB-ERF regulatory modules may represent a conserved mechanism governing pear aroma formation, while species-specific differences in expression levels and target gene preferences contribute to the diversity of aroma profiles among pear cultivars.

Current research has found that the synthesis of the four aromatic compounds mentioned above is closely related to fatty acid metabolic pathways. Ethyl acetate and ethyl 2-methylbutyrate belong to the branched-chain ester class; their synthesis depends on acyl-CoA substrates generated by the branched-chain amino acid elongation pathway, which are then catalyzed by alcohol acyltransferase (AAT) to form volatile esters [35]. 1-Hexanol is synthesized from fatty acids via the hydroperoxide cleavage step catalyzed by lipoxygenase (LOX) and the reduction step catalyzed by alcohol dehydrogenase (ADH) [9]. As a long-chain alkane, the synthesis of hexadecane is closely linked to the metabolic pathways of hydrocarbons derived from fatty acids [3]. Thus, it can be seen that the fatty acid metabolic pathway is the common upstream rate-limiting step in the synthesis of the aforementioned aromatic compounds. Within the fatty acid metabolic network, fatty acid desaturase (FAD) serves as a key node regulating fatty acid composition and unsaturation; it not only influences membrane lipid fluidity and fruit texture but also indirectly regulates the production of volatile aldehyde, alcohol, and ester precursors by altering the composition of free fatty acids [9]. This study demonstrated through a dual-luciferase reporter assay that PuMYB44 and PuERF118 can independently bind to and activate the transcription of PuFAD8; furthermore, when co-expressed, their transcriptional activation of *PuFAD8* is significantly enhanced (Figure 5f,g). However, the study has not yet validated the predicted cis-acting elements on the PuFAD8 promoter through site-directed mutagenesis; therefore, the specific binding sites of PuMYB44 and PuERF118 on the PuFAD8 promoter remain to be further refined (Figure 5d).

It is worth noting that in transient expression experiments, we found that the PuMYB44-PuERF118 regulatory module not only promotes the accumulation of aromatic compounds but also significantly induces anthocyanin biosynthesis in pear peel (Figure 3a). This synergistic regulatory pattern of color and aroma traits holds significant biological importance; previous studies have shown that plant color and aroma traits typically coevolve to effectively attract pollinators and promote seed dispersal [36]. For example, in roses, RcMYB1 simultaneously regulates the synthesis of anthocyanins and the accumulation of volatile metabolites [37]. In tomatoes, SlMYB75 promotes anthocyanin accumulation by activating the expression of downstream genes such as LOXC, AADC2, and TPS, thereby enhancing the production of volatile aromas in tomato fruits [38]. In this study, transient overexpression of either the PuMYB44 or PuERF118 gene alone induced pigment accumulation in pear fruit peel, and the regulatory effect was even more pronounced when the two genes were co-expressed (Figure 3a). However, whether this complex exerts its effect by synergistically regulating key structural genes in the anthocyanin biosynthesis pathway, as well as its specific molecular role in the synergistic regulatory network governing color and aroma, requires further verification.

**Figure 6:**
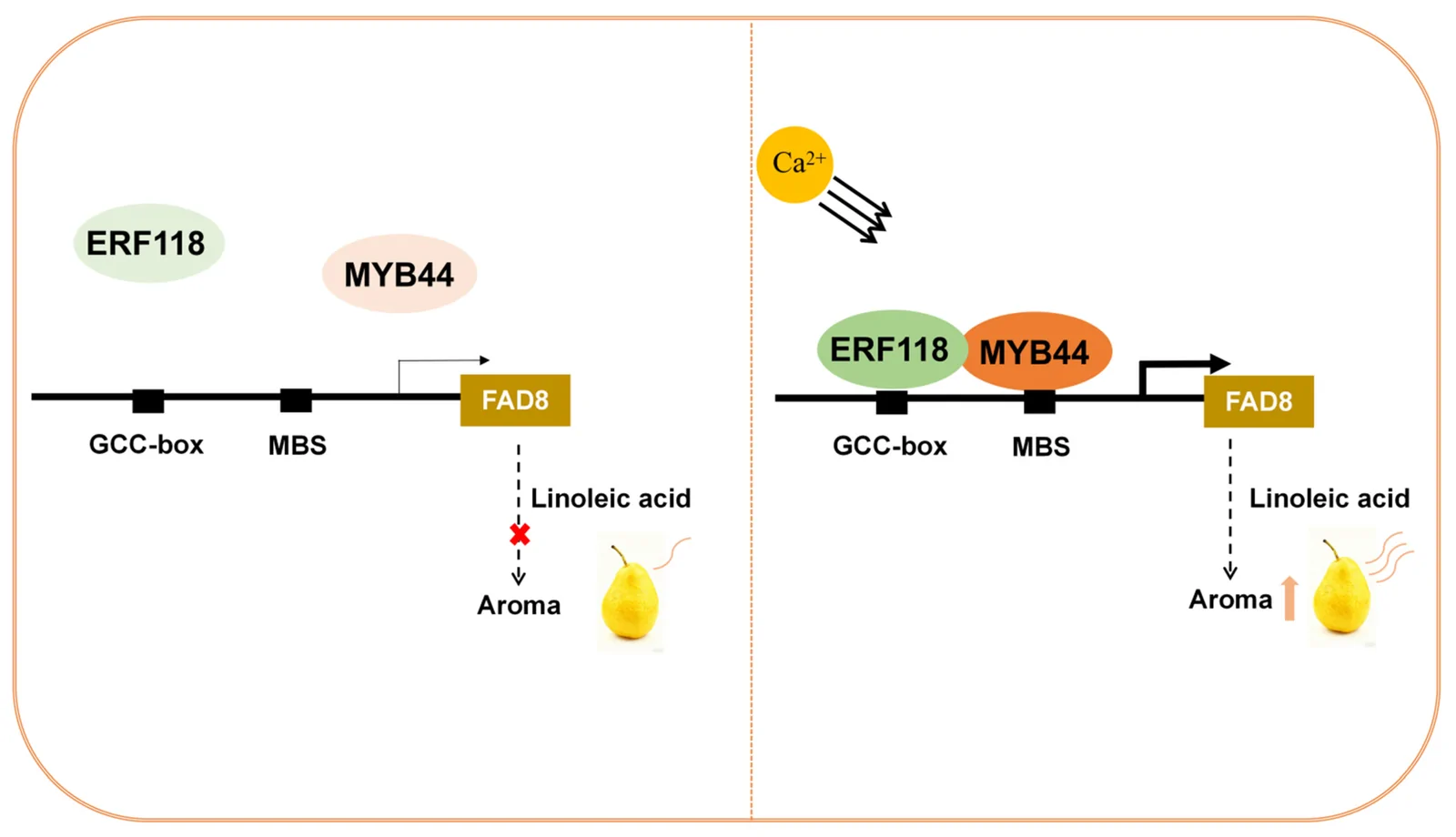
Model. Calcium signaling mediates the expression of PuMYB44 and PuERF118, which subsequently bind to the *PuFAD8* promoter, respectively, thereby activating *PuFAD8* expression. Upregulated *PuFAD8* promotes the desaturation of fatty acid substrates, ultimately conferring aroma in pear fruit.

Overall, this study revealed a calcium-signal-mediated PuMYB44-PuERF118 molecular module that drives the biosynthesis of aroma compounds in pear fruit by synergistically activating *PuFAD8* expression (Figure 6). These findings not only deepen our understanding of the transcriptional networks regulating pear aroma formation but also provide valuable candidate genes for breeding high-quality pear varieties with superior flavor and visual quality.

## 4. Materials and Methods

### 4.1. Plant Materials and Growth Conditions

From the ‘Nanguo’ pear (*Pyrus ussuriensis*) experimental orchard, pear trees with similar growth conditions were selected and divided into two groups. One group was treated by spraying a 4% (*w*/*v*) calcium chloride solution onto the fruit surface, while the other group was sprayed with water as a control (CK). Samples of the treated pears were collected five days before harvest (F5B), at harvest (F5), and five days after harvest (F5L) for transcriptomic sequencing. In addition, mature ‘Nanguo’ pears were harvested for transient transfection experiments. The ‘Daxiangshui’ and the ‘Hwangkeum’ used for comparative validation were harvested from an orchard in Shandong Province, China, at the same stage of ripeness. The samples were immediately frozen in liquid nitrogen and stored in a freezer (Zhejiang Heli Refrigeration Equipment, Jinhua, China) at −80 °C for use in subsequent experiments. The *Nicotiana benthamiana* plants used in this luciferase complementation assay were grown in the greenhouse of the School of Food and Biological Engineering at Hefei University of Technology under conditions of 25 °C and a 16-h photoperiod.

### 4.2. Gene Cloning and Vector Construction

Using cDNA obtained from *Pyrus ussuriensis* as a template, we designed specific primers based on gene sequences obtained from PGDB (http://pyrusgdb.sdau.edu.cn/, accessed on 12 March 2026) and amplified the CDS sequences of PuMYB44, PuFAD8, and PuERF118. Subsequently, we cloned the CDS sequences of these genes into the corresponding empty vectors and transformed them into DH5α (Yeasen Biotechnology, Shanghai, China), (heat shock at 42 °C for 45s, incubation on ice for 2 min, suspension culture at 37 °C for 1-h). The plates were then incubated upside down on solid LB medium at 37 °C for 16-h to obtain DH5α cells successfully transformed with the recombinant plasmids. Finally, the successfully constructed recombinant plasmids were transformed into GV3101 (Yeasen Biotechnology, Shanghai, China), (5 min in an ice bath, 5 min in liquid nitrogen, 5 min at 37 °C, 5 min on an ice bath, 3 h of suspension culture at 28 °C, and finally 2–3 days of inverted growth on solid medium containing antibiotics). The GV3101 cells containing the recombinant plasmid were flash-frozen in liquid nitrogen under a GV3101: glycerol ratio of 1:1 and stored at −80 °C for subsequent experiments.

### 4.3. Transient Overexpression in Pear Fruit

The previously obtained pSAK277 vectors—one containing an empty vector and the others carrying PuMYB44, PuERF118, and PuFAD8, respectively—were transformed into Agrobacterium (see Section 4.2). We then cultured the obtained Agrobacterium strains at 28 °C for 1–2 days, added an infection solution (10 mM MES, 10 mM MgCl_2_, 0.2 µM Acetosyringone in water), and used a spectrophotometer to measure the OD_600_ at 0.6–0.8. After 2–3 h of suspension culture, Agrobacterium suspensions containing the empty vector, PuMYB44, and PuMYB44 + PuERF118 were injected into pear pulp using a 1 mL sterile syringe. The treated pear fruits were cultured at 25 °C in the dark for 1 day, followed by 5 days of cultivation at 25 °C under light. Fruit pulp tissue was collected for volatile organic compound (VOC) analysis and quantitative real-time fluorescent quantitative PCR (qRT-PCR) analysis (Roche, Basel, Switzerland). A total of three treatment groups were established: an empty vector control group, a PuMYB44 single-gene overexpression group, and a PuMYB44 + PuERF118 dual-gene co-overexpression group. Each treatment group consisted of 30 fruits, with three biological replicates. The primer sequences used in the experiment are detailed in Table S1.

### 4.4. Volatile Organic Compound Detection via HS-SPME-GC-MS

Two grams of pear pulp were homogenized, transferred to 20 mL headspace vials with saturated NaCl solution, and incubated at 60 °C for 30 min; volatile compounds were then extracted using a 50/30 μm DVB/CAR/PDMS SPME fiber (Supelco, Bellefonte, PA, USA). GC-MS analysis was performed on Agilent 7890A GC coupled with 5975C MS detector (Agilent Technologies, Santa Clara, CA, USA). VOCs were identified by matching mass spectra with NIST 14 library, quantified via an internal standard (2-octanol), and classified into esters, aldehydes, alcohols, and terpenoids. The total volatile content was calculated as μg·g^−1^ FW. Each sample had three biological replicates.

### 4.5. Dual-Luciferase Reporter Assay

The 2000 bp upstream promoter sequence of *PuFAD8* was amplified and inserted into the pGreenII 0800-LUC vector (Yeasen Biotechnology, Shanghai, China). The transcription factors were cloned into the previously constructed pSAK277-GFP recombinant plasmids carrying the target genes. The recombinant plasmids were co-transformed into Agrobacterium tumefaciens strain GV3101 together with the pSoup helper plasmid. After large-scale culture of the Agrobacterium strains carrying the recombinant plasmids in liquid LB medium, the cells were collected by centrifugation at 5000 rpm for 10 min, resuspended in infiltration buffer, and the transcription factor and promoter constructs were mixed at a ratio of 9:1. The mixture was injected into young tobacco leaves for transient co-expression analysis. Transcriptional activation activity was measured as the ratio of firefly luciferase to Renilla luciferase using a Dual-Luciferase Reporter Assay System (Yeasen Biotechnology, Shanghai, China). For in vivo LUC imaging, D-luciferin potassium salt (1 mmol·L^−1^, dissolved in 10 mmol·L^−1^ sodium phosphate buffer) was injected into the injection sites 48 h after agroinjection. After 5 min in the dark to reduce background fluorescence, luminescence images were acquired using a live-image plant imaging system with an exposure time of 3–5 min (Biolight Biotechnology, Guangdong, China). The false-color scale represents luminescence intensity. The primer sequences used in the experiment are detailed in Table S2.

### 4.6. Quantitative Real-Time PCR (qRT-PCR)

Total RNA extracted from pear pulp was reverse-transcribed into cDNA, and quantitative primers were designed online based on the CDS sequences using the IDT RealTime PCR tool (https://sg.idtdna.com/scitools/Applications/RealTimePCR/default.aspx, accessed on 19 March 2026) (Table S1). Subsequently, RT-qPCR was performed using the Taq SYBR Green qPCR Premix Kit (Roche, Basel, Switzerland) in a 20 μL reaction system. The amplification protocol is as follows: 30 s of pre-denaturation at 95 °C, followed by 40 amplification cycles (each cycle consisting of 10 s of denaturation at 95 °C and 30 s of annealing/extension at 60 °C). Relative gene expression levels were calculated using the 2^−ΔΔCt^ method. All data and error bars are based on three biological replicates. The primer sequences used in the experiment are detailed in Table S3.

### 4.7. Yeast Two-Hybrid Assay (Y2H)

Following the protocol for the Matchmaker Gold yeast two-hybrid (Y2H) system (Clontech, website: http://www.clontech.com/, accessed on 30 March 2026), Y2H assays were conducted to investigate protein–protein interactions. The CDS sequences of PuERF118 and PuMYB44 were cloned into the pGADT7 and pGBKT7 vectors, respectively, and co-transformed into the yeast strain Y2HGold. Colonies harboring both recombinant plasmids were selected on SD/-Leu/-Trp medium and then validated on SD/-Leu/-Trp/-His/-Ade medium. Co-transformation experiments using pGADT7-T and pGBKT7-Lam, as well as pGADT7-T and pGBKT7-53, were used as negative and positive controls, respectively. The primer sequences used in the experiment are detailed in Table S4.

### 4.8. Luciferase Complementation Assay

Healthy *Nicotiana benthamiana* plants that had been growing for 4–5 weeks and were of uniform size were selected. Bacterial suspensions carrying cluc-PuMYB44 and nluc-PuERF118 were mixed in a 1:1 ratio to form the experimental group. The following negative control groups were also established: (1) cluc-PuMYB44 + nluc; (2) cluc + nluc-PuERF118; (3) nluc + cluc. Using a 1mL sterile syringe, the mixed bacterial suspension was slowly injected into the lower epidermis of the tobacco leaves. Three to four leaves per tobacco plant were injected, and three sets of tobacco plants were used for each treatment. After injection, the plants were placed in a greenhouse at 25 °C with a 16-h light/8-h dark cycle and cultured for an additional 48–60 h. Live imaging was then performed. Prior to imaging, a 1 mmol·L^−1^ solution of D-luciferin potassium salt (dissolved in 10 mmol·L^−1^ sodium phosphate buffer, pH 8.0) was injected into the injection site. The tobacco plants were then placed in the dark for 5–10 min to reduce background fluorescence. Chemiluminescence was detected using a live-image plant imaging system, with an exposure time set to 3–5 min and images acquired in medium-resolution mode. Quantitative analysis of the luminescent regions was performed using Living Tanon GIS software (version 3.6.7.2), with average radiance (p·s^−1^·cm^−2^·sr^−1^) as the quantitative measure of interaction intensity. The primer sequences used in the experiment are detailed in Table S5.

### 4.9. Phylogenetic Tree Construction and Analysis of Conservative Domains

After obtaining the coding sequence of the target gene from PGDB (http://pyrusgdb.sdau.edu.cn/index.html, accessed on 12 March 2026), a phylogenetic tree was constructed using the One Step Build an ML tree function in Tbtools, and conserved domain analysis was performed using ESPript (https://espript.ibcp.fr/ESPript/cgi-bin/ESPript.cgi, accessed on 14 March 2026).

### 4.10. Statistical Analysis

All experimental data were analyzed using SPSS 26.0 software. One-way analysis of variance (ANOVA) followed by Duncan’s multiple range test was used to compare significant differences between treatments (*p* < 0.05). Graphs were generated with Origin 2025 (version 10.2.5.212) and sequence alignments were visualized with MEGA 11 (version 11.0.13) and DNAMAN (version 10.0.2.100).

### 4.11. Accession Numbers

Sequence data from this article can be found in the genome database for the PGDB (pyrusgdb.sdau.edu.cn) under the following accession numbers: *PuMYB44* (Pbr025199.1), *PuERF118* (Pbr028836.1), *PuFAD2* (Pbr035830.1), *PuFAD3* (Pbr033732.1), *PuFAD8* (Pbr005591.1), *PuLOX5* (Pbr008100.1), *PuLOX21* (Pbr004967.1), and *PuSCD* (Pbr005674.1).

## Data Availability

Data are contained within the article or Supplementary Materials.

## Supplementary Materials

The following supporting information can be downloaded at: https://www.mdpi.com/article/10.3390/ijms27188070/s1.
