# Supplementary material for: Calcium-Triggered PuMYB44-PuERF118 Cascade Activates PuFAD8 to Promote Aroma Biosynthesis in Pear Fruit
Source: Int J Mol Sci. 2026 Sep 10;27(18):8070. doi: 10.3390/ijms27188070 (PMC13607563; doi:10.3390/ijms27188070)
Supplement: Supplementary file 1 [file ijms-27-08070-s001.zip › ijms-4504062-supplementary.pdf]

International Journal of
Molecular Sciences

MDPI

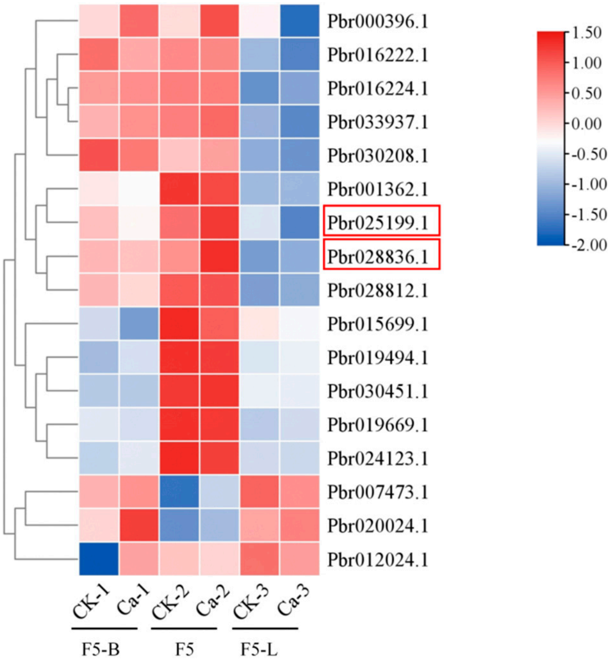

Figure S1. Heatmaps of the expression of aroma-related candidate genes in 'Nanguo' pears under calcium treatment and control conditions. F5B, F5, and F5L represent five days before harvest, the harvest date, and five days after harvest, respectively; red and blue represent high (1.5) and low (−1.5) expression values, respectively. The red rectangles highlight two candidate transcription factor genes: *PuMYB44* (Pbr025199.1) and *PuERF118* (Pbr028836.1).

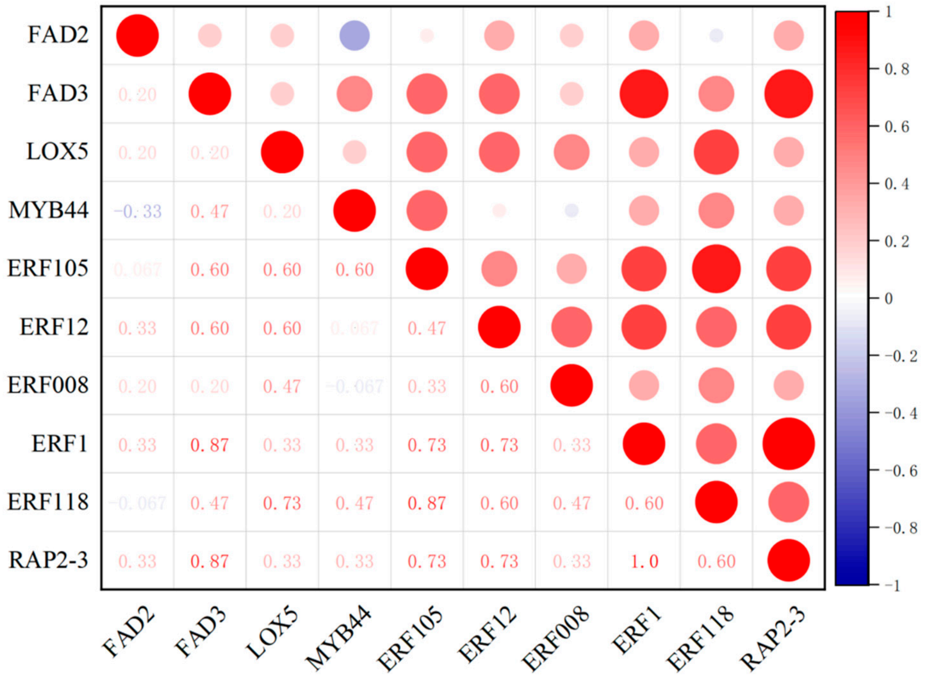

Figure S2. Heatmap showing the Pearson correlation coefficients for genes associated with the synthesis of aromatic compounds in the 'Hwangkeum' and 'Daxiangshui' pears, as well as the expression

levels of PuMYB44 and PuERF118. The size of the circles represents the absolute value of the correlation coefficient, and the color scale ranges from dark blue (-1, negative correlation) to dark red (1, positive correlation).

Table S1. The primer sequences for transient expression experiments

pSAK277-PuERF118-F	ACTAGTGGATCCAAAGAATTCATGGCTGTGCCTCGGAAAC
pSAK277-PuERF118-R	TCATTAAAGCAGGACTCTAGATTATTGGCATACTATGTTGAGGGG
pSAK277-PuMYB44-F	ACTAGTGGATCCAAAGAATTCGACGCACAATCCCACTATCC
pSAK277-PuMYB44-R	TCATTAAAGCAGGACTCTAGATTGATAATCATAACGATTAGCATTAC

Table S2. The primer Sequences for the Dual-Luciferase Assay

0800-PuFAD8-F	gtcgacggtatcgataagcttGCGAGTTTATTTTCTAAAAGATCAACC
0800-PuFAD8-R	cgctctagaactagtggatccAGCACACTATGTTGAAAATTACATGG
PuERF118-AD-F	gccatggaggccagtgaattcATGGCTGTGCCTCGGAAAC
PuERF118-AD-R	cagctcgagctcgatggatccATTGGCATACTATGTTGAGGGG
PuMYB44-AD-F	gccatggaggccagtgaattcGACGCACAATCCCACTATCC
PuMYB44-AD-R	cagctcgagctcgatggatccGATAATCATAACGATTAGCATTAC

Table S3. Primer sequences for RT-qPCR analyze the aroma-related genes in pears

PuTubulin-F	GGGCTTTGCTCCTCTTACTT
PuTubulin-R	GATCAGCAGCACACATCATATTC
PuSCD-F	TGCCACCGATGAGGAAGA
PuSCD-R	CGAGCCTAACCGCAACAA
PuFAD2-F	CCATTGCCTCCATCCTTTACT
PuFAD2-R	AGAACACAGCCCTGAACATAC
PuFAD3-F	AGAAGTCCAGGGAAGAAA
PuFAD3-R	CATCATAGTCCAGCAAGTAG
PuFAD8-F	AAGAGGAGCATCCCAGATAGA
PuFAD8-R	GCTCGCCAACACAAACATAAA
PuLOX5-F	GCAAGAGAGGAATGGCAGTT
PuLOX5-R	GTCCTGGAGTTCAAAATCACCT
PuLOX21-F	GACTACTTTCACCATCCCAGC
PuLOX21-R	CAACCCACTTGGTGTCTCTG
PuERF1-F	GGTACCAACCACACCATCAA
PuERF1-R	CGTATCTCAGCTGCCCATTTA
PuERF118-F	GATTCCGACTCTGTCGTTTCTC
PuERF118-R	GGCAGTCTCCTTCTCCAAATC
PuRAP2-3-F	GAGTTTCCAGACCGGTTCTTAC
PuRAP2-3-R	GAACGACTCCAAGCTCCATATC
PuMYB44-F	GACTCGCTCCAGAAGCTGGT
PuMYB44-R	AGAGGCGGCTGCTCGTGGTC
PuDFR-F	GGTTTCATCGGCTCTTGGC
PuDFR-R	CCTTCTTCTGATTCGTGGGGT
PuANS-F	GAGCAGAAGGAGAAGTAT
PuANS-R	ACAGTGGAAGAAGTAGTC

Table S4. The primer Sequences for Yeast Two-Hybrid Assays

PuMYB44-BD-F	tcagaggaggacctgcatatgGACGCACAATCCCACTATCC
PuMYB44-BD-R	ctagttatgcggccgctgcagGATAATCATAACGATTAGCATTAC
PuERF118-AD-F	gccatggaggccagtgaattcATGGCTGTGCCTCGGAAAC
PuERF118-AD-R	cagctcgagctcgatggatccATTGGCATACTATGTTGAGGGG

Table S5. The primer Sequences for the Luciferase Complementation Assay

cluc-PuMYB44-F	CCGGGGCGGTACCCGGGATCCGACGCACAATCCCACTATCC
cluc-PuMYB44-R	ACGAAAGCTCTGCAGGTCGACGATAATCATAACGATTAG-CATTAC
Nluc-PuERF118-F	CGAGCTCGGTACCCGGGATCCATGGCTGTGCCTCGGAAAC
Nluc-PuERF118-R	ATTTCCCGGGAAGCTGCTAAATTGGCATACTATGTTGAGGGG
